# Interstitial lung disease of infancy caused by a new *NKX2‐1* mutation

**DOI:** 10.1002/ccr3.901

**Published:** 2017-04-04

**Authors:** Khalid H. Safi, John A. Bernat, Catherine E Keegan, Ayesha Ahmad, Marc B. Hershenson, Manuel Arteta

**Affiliations:** ^1^ Department of Pediatrics and Communicable DiseasesUniversity of Michigan Medical SchoolAnn ArborMichiganUSA

**Keywords:** Hypothyroidism, interstitial lung disease, thyroid transcription factor‐1

## Abstract

Patients with personal or family history of congenital hypothyroidism, and/or neurological findings that also have chronic respiratory symptoms may have a mutation in the NKX2.1 gene as the unifying cause of their disease. Brain–lung–thyroid disease is the ensuing condition, which although rare, needs to be part of the differential diagnosis**.**

## Introduction

The *NKX2‐1* is a gene that encodes the thyroid transcription factor 1, a member of the homeodomain‐containing family of transcription factors. During human development, *NKX2‐1* is expressed early in fetal life in the thyroid bud and prosencephalon, and later in the lung epithelium 1, 2 *NKX2‐1* is critical for the expression of surfactant proteins A, B, and C, as well as ABCA3 3, 4, 5, a lipid transporter in pulmonary surfactant biogenesis. Haploinsufficiency for *NKX2‐1* due to either complete gene deletions or loss‐of‐function mutations results in brain–thyroid–lung syndrome with affected individuals having variable degrees of pulmonary disease (neonatal respiratory distress syndrome or children's interstitial lung disease), hypothyroidism, and neurologic abnormalities (typically benign hereditary chorea) 6, 7, 8, 9, 10, 11. However, the classical triad is not always present and severity of the thyroid, neurological, and pulmonary disorders vary widely 8, 12. In this report we describe a family of two siblings, and their mother with a *NKX2‐1* mutation that has not previously been associated with lung disease. The mother signed an informed consent for the publication of these cases.

## Case Presentations

**Figure 1:**
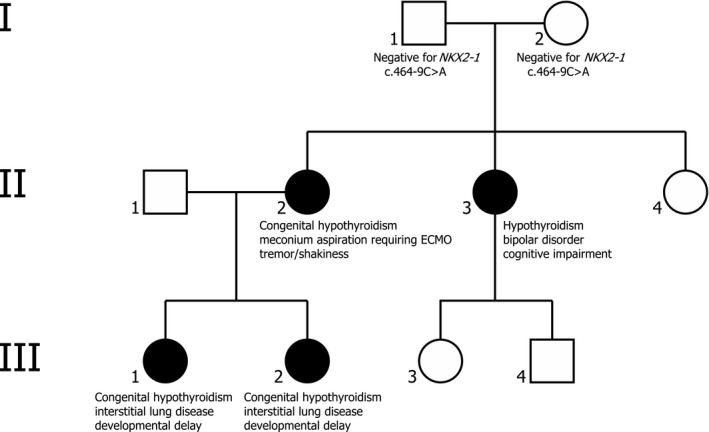
Pedigree. Filled symbols represent individuals testing positive for NKX2‐1 c.464‐9C>A.

The mother of the two siblings described in this report has congenital hypothyroidism (Fig. 1, II‐2). She required extracorporeal membrane oxygenation (ECMO) therapy in the neonatal period for respiratory failure due to meconium aspiration, she was discharged home without supplemental oxygen and has not had any significant respiratory illnesses since then. She also has gait ataxia and tremors which were attributed to brain injury following ECMO therapy. The maternal aunt (Fig. 1, II‐3) has hypothyroidism but no respiratory or neurologic disease. Both the mother and maternal aunt were found to carry the same *NKX2‐1* gene variant as the proban case. The maternal grandparents have no history of thyroid, neurologic, or respiratory disease, and they do not carry this gene variant.

### Case 1

Our first encounter with the family was with the oldest child (Fig. 1, III‐1). She was born full term without complications. Her newborn screen was positive for hypothyroidism, and she was started on thyroid hormone replacement. At 4 months of age, she was admitted for management of preseptal cellulitis. During that admission she was found to have mild tachypnea, subcostal retractions, bilateral lung crackles, and an oxyhemoglobin saturation ranging between 83% and 90% on room air. Initial radiograph of the chest was unremarkable. Further work up included high resolution computed tomography (HRCT) of the chest, bronchoscopy, echocardiogram, sleep study, video swallow study, and brain MRI. The HRCT of the chest showed only some dependent atelectasis, but normal lung parenchyma, the sleep study revealed mild obstructive hypopnea that improved with oxygen supplementation but no hypoventilation, and the video swallow study revealed aspiration of thin liquids. The child was discharged home on oxygen via nasal cannula (1.5 L/min), thickened formula, and thyroid hormone replacement. *NKX2‐1* gene analysis was reported as heterozygous for an intronic variant, c.464‐9C>A. This mutation was considered a variant of uncertain significance as it had not been previously reported in patients or unaffected individuals; however, splice site calculators indicated a potential reduction in correct splicing.

Over the next few months, her oxygen requirement increased to 2 L/min. At 12 months the age she was admitted with severe respiratory distress requiring endotracheal intubation and mechanical ventilation due to human metapneumovirus infection. She developed severe ARDS requiring 2 weeks of ECMO therapy. She recovered from this acute illness and was discharged home with supplemental oxygen at 2.5 L/min via nasal cannula and nasogastric tube feeding due to oral aversion.

She was admitted a third time at age of 20 months with respiratory syncytial virus (RSV) bronchiolitis and severe respiratory distress requiring endotracheal intubation and high frequency mechanical ventilation. Three weeks later she was discharged with an increased oxygen requirement of 3 L/min. A repeat swallow study during this admission was normal. The child was seen in pulmonary clinic at 22 months of age. At that time, respiratory rate was 70/min on 2 L/min supplemental oxygen, oxyhemoglobin saturation on room air was 82%, and lung examination showed severe retractions, and diffuse crackles. A repeat HRCT of the chest showed diffuse mixed ground‐glass and ill‐defined nodular opacities scattered throughout both lungs. Treatment was begun with triple anti‐inflammatory therapy consisting of prednisolone (2 mg/kg every other day), azithromycin 80 mg three times a week, and hydroxychloroquine (6 mg/kg/day). Two months later, the child showed significant improvements in growth, respiratory rate, and lung examination. One year after initiating chronic anti‐inflammatory therapy, prednisolone dose had been weaned to 0.7 mg/kg every other day, oxygen therapy remained at 2 L/min, her oxyhemoglobin saturation had improved to 90% on room air, respiratory rate was 42/min, there were no retractions, lung examination showed only sparse crackles at the bases, and she had not required hospitalizations.

### Case 2

The younger sister (Fig. 1, III‐2) was born to the same parents at term without complications. She was also diagnosed with congenital hypothyroidism by newborn screening and was started on thyroid hormone replacement therapy soon after birth. She did not have any respiratory problem until 4 months of age when she developed RSV bronchiolitis. During that illness she was found to be hypoxemic and required oxygen therapy for a few days, but discharged home on room air and with a normal respiratory examination. Genetic testing for *NKX2‐1* demonstrated the same variant as her older sister. She was admitted to a local hospital at the age of 7 months with pneumonia and was treated with antibiotics, during that admission she again required oxygen supplementation. Upon follow‐up in the pulmonary clinic at 8 months of age, she was started on maintenance prednisolone therapy of 2 mg/kg/day, which was weaned off slowly without development of respiratory symptoms.

## Discussion

The individuals from the family described in this report possess a heterozygous *NKX2‐1* gene variant (c.464‐9C>A; p.Ile155Thrfs*286). Because the variant was located within an intron and had not been previously linked to brain–lung–thyroid syndrome, it was initially reported as a variant of uncertain significance. However, the same *NKX2‐1* variant was subsequently reported to be linked to benign hereditary chorea in a Japanese family 13. This family included six individuals in three generations presenting with choreic involuntary movements in childhood, without thyroid or respiratory problems. Further study of this variant found that it activates a cryptic splice acceptor site, resulting in the insertion of seven nucleotides in the transcript. This is predicted to cause a change in the reading frame resulting in an abnormal protein with amino acid substitutions beginning at amino acid 155. To our knowledge, this *NKX2‐1* mutation has not been reported in association with lung disease previously.

A molecular defect in the *NKX2‐1* gene was first identified in 1998 in a patient with neonatal thyroid dysfunction and respiratory failure, who later developed hypotonia and truncal ataxia 7. Subsequently other cases were described, and the term “Brain–Thyroid–Lung syndrome” was coined in 2005 8, 9, 10, 11. Since then this term has become widely used to describe patients who have neurological, thyroid, and/or lung disorders in association with *NKX2‐1* gene mutations 8, 12. The classical triad of this syndrome is not always present, as can be appreciated in the family described in this report; and the severity of neurological, thyroid, and lung compromise is also variable 10. The lung disease may present as respiratory distress syndrome, interstitial lung disease, pulmonary fibrosis, or recurrent respiratory tract infections 11, 14, 15, 16, 17, 18.

In the family we describe in this report, we have not only observed variation in the organ systems involved, but also a variable degree of lung disease, this is consistent with the findings of Hanvas et al. in a case series of 21 patients with different *NKX2‐1* mutations and variable degree of lung disease recently reported 10. The same observation was noted with regard to thyroid and neurological involvement by Gras et al., who concluded that there were no genotype–phenotype correlations, and that there was phenotype variability in individuals with the same mutation 14. Hanvas et al. also reported that recurrent pulmonary infections were a prominent feature in patients with *NKX2‐1* mutations 10, this phenomenon can also be appreciated in the proband case of our report.

The patients’ maternal grandparents both tested negative for the familial *NKX2‐1* variant, but had two children (the patients’ mother and maternal aunt) who tested positive for the variant. It is then likely that one of the grandparents is mosaic for the variant, which was not detectable in DNA extracted from a blood sample. However, the possibility of misattributed parentage was not specifically excluded.

Hydroxychloroquine has been successfully used in the treatment of both surfactant protein C and ABCA3 deficiencies 19, 20. Since the transcription factor *NKX2‐1* regulates the expression of both surfactant protein C and ABCA3, we reasoned that hydroxychloroquine would be useful in the treatment of brain–lung–thyroid disease associated with an *NKX2‐1* mutation. Therapy with hydroxychloroquine, along with azithromycin and prednisolone, was associated with significant clinical improvement in the sibling with life‐threatening lung disease. As far as we are aware, this is the first report of seemingly successful treatment of *NKX2‐1* associated lung disease with this combination therapy, but given the variable phenotypic manifestations of lung involvement in infants with this condition, and the fact that our patient was recovering from a severe pulmonary viral infections, our ability to state with a high degree of certainty that this drug therapy improved the underlying lung disease is limited. On the other hand her symptoms responded promptly, and she has been doing well since then suggesting that this intervention has played a role.

## Conclusion

Mutations in the *NKX2‐1* gene result in variable disease phenotypes. The classical triad of brain–thyroid–lung syndrome is not always present, and different presentations may result from same mutations. A high index of suspicion should be maintained in patients with lung disease of unknown cause, especially if there is associated neurological or thyroid disease or a family history of such disorders. In this report, we show that the c.464‐9C>A variant in the *NKX2‐1* gene can be associated with life‐threatening infantile lung disease, and that treatment with hydroxychloroquine, azithromycin, and prednisolone might be helpful in such cases.

## Authorship

KHS, MBH, and MA: drafted the manuscript and contributed to treating the patients. JAB, CK, and AA: contributed to evaluating the patients and relatives, and critically revised and edited the manuscript. All authors have read and approved the final manuscript.

## Conflict of Interest

None declared.
